# Fatigue: a frequent and biologically based phenomenon in newly diagnosed celiac disease

**DOI:** 10.1038/s41598-022-11802-8

**Published:** 2022-05-04

**Authors:** Berit Mære Skjellerudsveen, Roald Omdal, Anne Kristine Hetta, Jan Terje Kvaløy, Lars Aabakken, Inger Marie Skoie, Tore Grimstad

**Affiliations:** 1 Department of Internal Medicine, Stavanger University Hospital, Pb. 8100, 4068 Stavanger, Norway; 2 Department of Mathematics and Physics, University of Stavanger, Stavanger, Norway; 3 Department of Research, Stavanger University Hospital, Stavanger, Norway; 4 Department of Transplantation Medicine, Rikshospitalet, Oslo University Hospital, Oslo, Norway; 5 Department of Clinical Science, University of Bergen, Bergen, Norway; 6 Department of Dermatology, Stavanger University Hospital, Stavanger, Norway

**Keywords:** Coeliac disease, Immunology, Fatigue

## Abstract

Fatigue is increasingly recognized as a major complaint in patients with chronic inflammatory and autoimmune diseases. Although fatigue is assumed to represent a significant problem in celiac disease, existing knowledge is scarce, and opinions are conflicting. This study aimed to investigate the prevalence and severity of fatigue in patients with newly diagnosed celiac disease and compare it with healthy control subjects. Ninety patients with newly diagnosed celiac disease were compared with 90 age- and sex-matched healthy subjects. The primary endpoints were fatigue severity as measured by: the fatigue Visual Analog Scale (fVAS), the Fatigue Severity Scale (FSS), and the inverted Vitality subscale of the MOS36 (SF-36vs). Higher scores indicate more severe fatigue. Clinically relevant fatigue was determined using predefined cut-off values. Secondary endpoints were the associations between fatigue, and sex, age, depression, pain, and selected biochemical variables. The median (IQR) fVAS-scores were 43.0 (18.0–64.5) in patients, and 9.0 (2.0–16.0) in the control group (*p* < 0.001); and the FSS scores 3.8 (2.0–4.8) in patients, and 1.4 (1.0–1.9) in control subjects (*p* < 0.001). Inverted SF-36vs scores had a mean (SD) value of 58.8 (23.6) in patients, and 29.7 (14.3) in healthy subjects (*p* < 0.001). The presence of clinically relevant fatigue ranged from 41 to 50% in patients. Increased fatigue severity was associated with female sex, younger age, and elevated pain and depression scores, but not with levels of selected biochemical variables, including hemoglobin. Fatigue is a severe and frequent phenomenon in patients with untreated celiac disease.

## Introduction

Fatigue may be defined as “an overwhelming sense of tiredness, lack of energy, and feeling of exhaustion”^1^. It is frequently observed in patients with chronic inflammatory and autoimmune diseases, cancer, and neurodegenerative diseases^2–5^. Many patients rate fatigue as one of their most severe and troublesome afflictions, as it affects emotional, physical, and social aspects of life, and is often dismissed by their treating physician^6–9^. Although fatigue as a phenomenon has gained increased attention in recent years, the mechanisms behind its generation and regulation are only beginning to be revealed.

A conceptual model for understanding fatigue is the “*sickness behavior response*”, an evolutionarily conserved survival enhancing strategy observed in animals and humans during states of infection or bodily harm^10,11^. The sickness behavior response encompasses several phenomena such as weariness; fatigue; lack of thirst, hunger, and grooming; as well as social withdrawal and depression^11^.

Sickness behavior is triggered by the activation of innate immunity and neuroinflammation. The proinflammatory interleukin (IL)-1β is produced and released by macrophages in inflammatory states and transported to the brain, inducing sickness behavior after binding to specific IL-1 receptors^7^. In both animal and human studies, infusion of inflammatory cytokines results in sickness behavior^12,13^; fatigue is a dominant phenomenon in this complex response. Accumulating evidence points to a genetic basis and complex molecular pathways that generate fatigue in the brain through neuronal signaling^14–16^. In addition, psychosocial factors such as depression, disturbed sleep, and pain all influence fatigue severity^17^.

Celiac disease is an autoimmune inflammatory disease of the small intestine, driven by an immune reaction to dietary gluten in genetically susceptible individuals^18^ and affecting approximately 1% of the population worldwide^19^. Traditionally, it was believed that celiac disease is confined to the intestine, with classical symptoms mainly related to malabsorption, such as diarrhea, stomach pain, and weight loss^18^. However, it has become evident that extraintestinal manifestations such as arthralgia, osteoporosis, anemia, and headaches are frequent, and may dominate or represent the only manifestation of the disease^18,20–22^. These extraintestinal manifestations may result from both aberrant immune responses and malabsorption^22^; extreme tiredness, and fatigue have been among the most commonly reported extraintestinal complaints^20,21,23^. This contrasts with the traditional concept of the disease, possibly explaining why current literature regarding fatigue in celiac disease is sparse and inconsistent^24,25^.

Previously only a few studies have been dedicated to explore fatigue in celiac disease, and in general the more phenomenologic issues have been focused on. We believe there is a knowledge gap regarding the more objective and biologically based factors of fatigue in celiac disease, and to obtain a better understanding of these issues, we investigated patients newly diagnosed with the disease, and compared them with healthy age- and sex-matched subjects.

## Methods

### Patient recruitment and diagnostic procedures

This study was performed at the Department of Gastroenterology, Stavanger University Hospital, and was designed as a single-center, cross-sectional, controlled study. Eligible patients were consecutively identified based on referral letters to the outpatient clinic. The inclusion criteria were: positive tests for anti-tissue transglutaminase-IgA antibodies (tTG-IgA) at time of referral, defined as levels ≥ 7 U/mL (normal: < 7); age ≥ 18 years; and being on a gluten-containing diet. Patients fulfilling these criteria were considered for participation, and eligible patients were included at the study visit. An upper endoscopy, four mucosal biopsies from the descending part of the duodenum, and two biopsies from the proximal duodenal bulb were performed for histopathological verification of celiac disease. Exclusion criteria were histopathological findings not consistent with celiac disease, inability to consent, and inability to adhere to the treatment protocol. Patients with findings consistent with celiac disease without villous atrophy in the biopsies (Marsh I and II histology) were also included, as guidelines at the time of inclusion used the term “probably celiac disease” for these cases^19^.

### Patient handling

Eligible patients were informed regarding the study and procedure, and provided written, informed consent before inclusion in the study. During the visit, baseline data, including demographics and clinical data, were registered; blood samples were taken before gastroscopy was performed. All data were electronically registered on an iPad case report form using the FileMaker Pro (Claris International) software.

### Healthy subjects

Ninety self reportedly healthy control subjects were included in the study. They were predominantly recruited from nonfamily acquaintances of patients with inflammatory bowel disease or psoriasis; approximately 1/3 were recruited from employees of the hospital and their acquaintances. The healthy control subjects were without any chronic illness. We did not perform any physical examination, blood tests or review of their medical records to verify that they were healthy.

### Matching procedure

**Figure 1:**
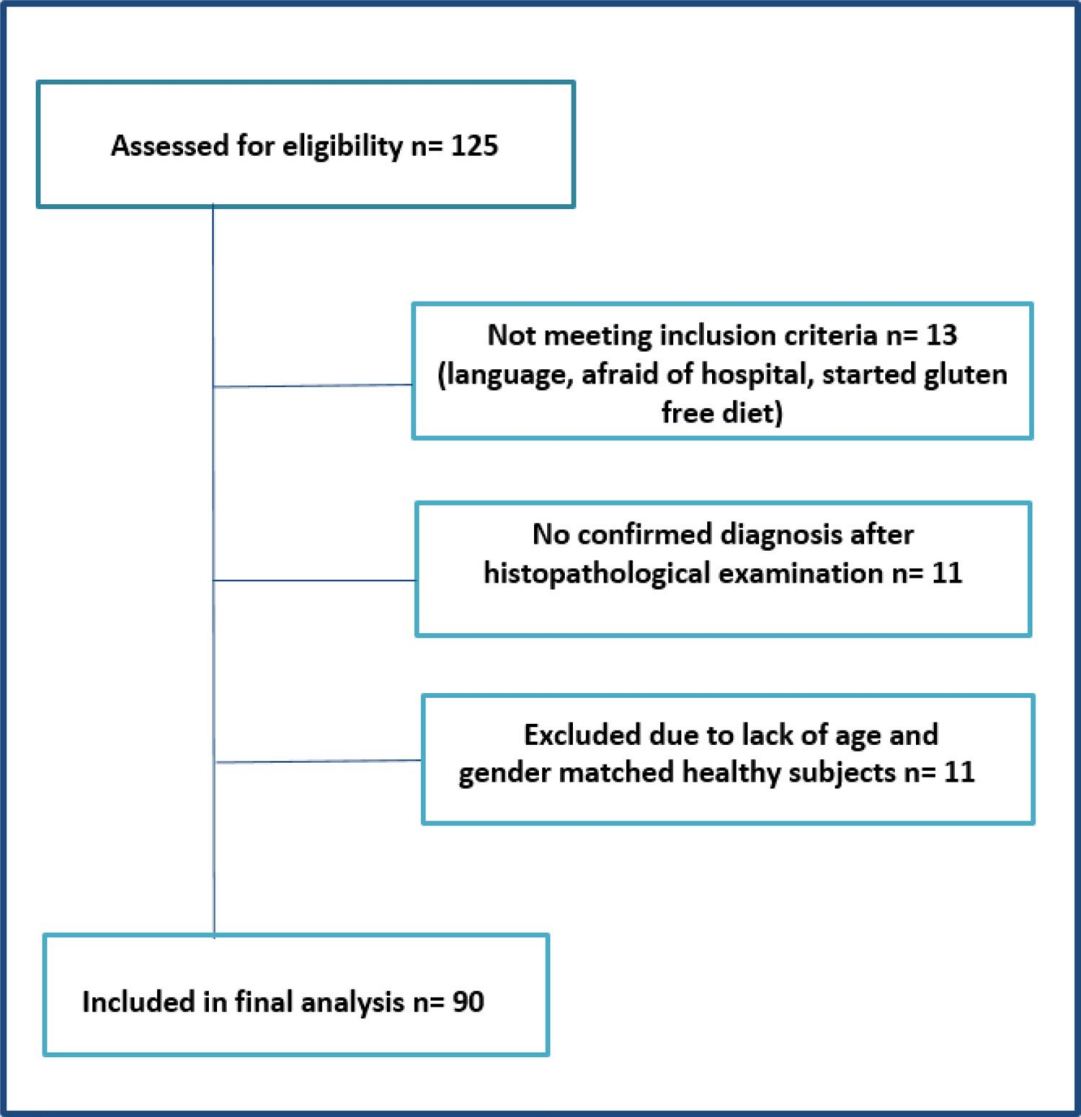
Flowchart of patient inclusion.

During the inclusion period between December 1, 2016, and September 30, 2018, 125 subjects were screened for participation; celiac disease was confirmed in 101 cases (80.8%). Eleven patients were excluded, as no control subjects fulfilled the matching criteria, i.e., sex and age +/− 5 years; thus, a total of 90 matching pairs were included for further study (Fig. 1).

### Demographic and clinical data

Age, sex, and body mass index (BMI) were registered for all study participants. The initial symptoms and signs that led to contact with the health service, later resulting in a diagnosis of celiac disease, were registered upon study visit based on the following predefined items: abdominal discomfort, diarrhea, stomach pain, weight loss, and weariness, there was also a possibility to add free text to further specify.

Concurrent autoimmune disease was registered based on patients’ reporting and medical files. Patients were dichotomized into “any other autoimmune disease” or “only celiac disease” based on the presence of one or more concomittant autoimmune disease.

### Blood tests

C-reactive protein (CRP), hemoglobin, folic acid, cobalamin, 25-OH vitamin D, tTG-IgA, and total IgA were measured in all patients at study inclusion and analyzed at the hospital’s routine laboratory. tTG-IgA analysis was performed with a fluorescence enzyme immunoassay, using EliATM CelikeyTM assays on a Phadia® 250 analyzer (Thermo Fischer Scientific). Ferritin levels measured within the last 3 months before inclusion were recorded. As ferritin is an acute phase protein, iron deficiency was defined as < 30 µg/L ferritin if CRP was < 5 mg/L, or < 100 µg/L ferritin if CRP was ≥ 5 mg/L, as recommended^26^. Anemia was defined as an Hb ≤ 13.4 g/dL for men, and ≤ 11.7 g/dL for women; cobalamin deficiency was defined as < 167 pmol/L, 25-OH vitamin D deficiency as < 50 nmol/L, and folic acid deficiency as < 6.3 nmol/L.

### Histopathological evaluation

Duodenal biopsies were obtained during upper endoscopy and were further prepared and examined at the hospital’s Department of Pathology. All biopsies were formalin-fixed, paraffin-embedded, stained with hematoxylin and eosin (H&E), and graded according to the modified Marsh-Oberhuber classification^27^.

### Fatigue assessment

Three generic and unidimensional fatigue instruments were used to grade fatigue severity: the fatigue Visual Analog Scale (fVAS), Fatigue Severity Scale (FSS), and Vitality subscale of the Medical Outcomes Study 36-Item Short-Form Health Survey (SF-36vs)^28–30^; these were completed by both patients and healthy control subjects under the instruction and supervision of a study nurse.

The fVAS is a single-item scale consisting of a 100 mm horizontal line with vertical anchors; the wording on the left end of the line (0 mm) is “no fatigue,” while the right end (100 mm) is “fatigue as bad as it can be”; the patient places a mark on the horizontal line to describe the degree of fatigue during the last week^29^. The cut-off for clinically relevant fatigue was previously defined as fVAS scores ≥ 50 mm; this was also applied in our study^3,5^.

The FSS is a questionnaire including nine statements regarding fatigue over the last two weeks; subjects are asked to rate their responses from 1 (completely disagree) to 7 (completely agree). The FSS score is reported as the mean score of the nine questions, ranging from 1 to 7; a score of ≥ 4 was previously used as a cut-off for fatigue, and was used in this study^30,31^.

The SF-36vs consists of four questions addressing energy and fatigue within the last 4 weeks, yielding score values between 0 and 100, where a higher score indicates less fatigue (and “higher vitality”)^32^; clinically relevant fatigue was defined by a score ≤ 35^5,33^. The registered SF-36vs scores were inverted to make data presentation more comparable with the other scales, as well as to improve visual presentations in the figures; an inverted SF-36vs score ≥ 65 thus represents clinically relevant fatigue.

### Depression and pain

The Hospital Anxiety and Depression Scale, Depression Subscale (HADS-D) was used to detect depressive mood in patients and healthy control subjects. The HADS-D consists of seven items, each answered with a number from 0 to 3^34^. An HADS-D score of ≥ 8 was previously used as a cut-off for depression^35^.

Pain was assessed using the bodily pain subscale (SF-36 pain) of the SF-36 questionnaire. The SF-36 pain consists of two items, assessing body pain intensity and the interference of pain with normal activities within the last 4 weeks. The scores range between 0 and 100, where higher scores indicate less pain. The pain scores were inverted to make data presentation more comparable with the other scales, as well as to improve visual presentation in the figures.

### Statistical analysis

The sample size was determined based on the assumption that fatigue severity and the ratio of patients experiencing clinically significant fatigue were similar to other inflammatory conditions, such as rheumatoid arthritis and primary Sjögren’s syndrome^4,36^. To detect a difference of 25 in the fVAS-scores between patients with celiac disease and healthy subjects, with a power of 0.8 and a significance level of 0.05, a minimum of 33 subjects would be required in each group. We chose to include more patients than the minimum number required as the study visits did not cause significant inconvenience to patients, and to ensure we had sufficient data for follow up studies. Continuous data normality was evaluated using the Shapiro–Wilk test. When pairwise comparing celiac disease patients with healthy subjects, normally distributed data were analyzed using the paired t-test, and non-normally distributed data using the Wilcoxon paired samples rank test.

Univariable linear regression analysis using fVAS, FSS, and inverted SF-36vs as the dependent variables was performed using the following independent variables: sex, age, body mass index, hemoglobin, folic acid, cobalamin, 25-OH vitamin D, tTG-IgA, ferritin, HADS-D, inverted SF-36 pain and “any other autoimmune disease”.

Independent variables from univariable regression analyses with a *p* value < 0.2 were used to develop multivariable regression models; we also included tTG-IgA due to its clinical significance. Backward and forward model selection was used to reach a final multivariable regression model. Analyses were performed using IBM SPSS statistical software (Version 24.0. IBM Corp.); a *p* value < 0.05 was considered statistically significant. For all regression models we report standardized beta coefficients, whose interpretation is the impact of a one standard deviation increase in the corresponding independent variable.

### Consent to participate

Written informed consent was obtained from all individual participants included in the study.

### Ethics approval

The study was approved by the regional ethics committee (REC West, Norway 2011/2631) and conducted in compliance with the principles expressed in the Declaration of Helsinki. The study is registered on ClinicalTrials.gov (NCT01551563).

## Results

### Demographics and clinical data

Characteristics of the study participants are given in Table 1. Two patients had anti-tTG-IgA values below 7 at V0, but these two patients had elevated anti-tTG-IgA at the time of referral and were therefore included. The two patients with Marsh 1 histological findings at diagnosis had normalization of anti-tTG-IgA and histology after one year of a gluten free diet, finally confirming the diagnosis.

**Table 1:** Baseline characteristics in 90 patients with newly diagnosed celiac disease and 90 healthy control subjects.

Characteristic	Patients (n = 90)	Controls (n = 90)
Age (years)	39.5 (18–72)	39.5 (18–71)
**Sex**		
Male	40 (44%)	40 (44%)
Female	50 (56%)	50 (56%)
BMI (kg/m^2^)	24.4 (14.7–39.0)	24.0 (18.0–39.5)
Ferritin (µg/L)	54.0 (6–443)	
Hb (g/dL)	14.1 (1.3)	
tTG-IgA (U/mL)	49.5 (5.0–141.0)	
Cobalamine (pmol/L)	289 (145–1400)	
Folic acid (nmol/L)	11.0 (3.4–39.0)	
25-OH Vitamin D (nmol/L)	66.0 (21.5)	
**Marsh classification**		
1	2 (2%)	
2	1 (1%)	
3a	16 (18%)	
3b	34 (38%)	
3c	37 (41%)	

Data are displayed as number (%), median (range), except for Hb and 25-OH Vitamin D; mean (SD).

*BMI* Body mass index, *Hb* Hemoglobin, *tTG-IgA* Anti-tissue transglutaminase IgA antibodies.

There were nine missing ferritin values, one missing hemoglobin value, one missing SF-36vs value. According to the definitions used, 27 (33%) patients were iron-deficient, 5 (6%) were anemic, 21 (23%) had low vitamin D levels, 13 (14%) had a folic acid deficiency, and 3 (3%) had a cobalamin deficiency.

Fifteen (17%) of the patients had one or more concurrent autoimmune disease. The registered concurrent autoimmune diseases were; thyroid disease (n = 7), diabetes type I (n = 4), psoriasis (n = 2), rheumatoid arthritis (n = 1), primary biliary cholangitis (n = 1) and dermatitis herpetiformis (n = 1).

### Symptoms and signs causing contact with the health service

The most frequently reported symptoms were abdominal discomfort, including nausea, borborygmi, and abdominal distention (Table 2); weariness was reported in 48% of patients. The classic characteristics of celiac disease were diarrhea in 40%, stomach pain in 33%, and weight loss in 10% of patients.

**Table 2:** Symptoms and signs causing contact with the health service in 90 patients with newly diagnosed celiac disease.

Symptoms and signs	No. of patients (%)
Abdominal discomfort (for example, nausea, borborygmi, abdominal distention, other discomfort)	48 (53%)
Weariness	43 (48%)
Diarrhea	36 (40%)
Stomach pain	30 (33%)
Weight loss	9 (10%)

### Severity and prevalence of clinically significant fatigue

**Figure 2:**
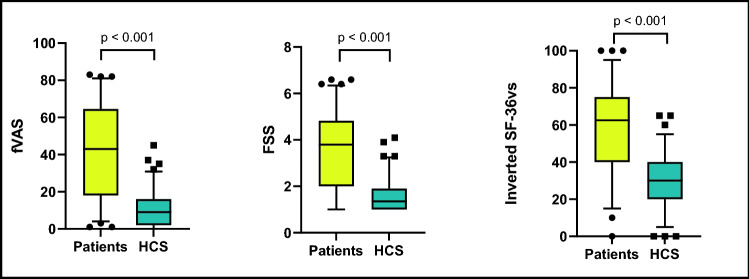
Fatigue measures using three different instruments in 90 patients with celiac disease and 90 healthy age- and sex-matched subjects. Box plots indicate median (line), interquartile range (box), and 5th to 95th percentiles (whiskers); dots represent outliers. To the right in the figure SF-36vs scores are inverted to ease visual interpretation. *fVAS* Fatigue visual analog scale, *FSS* Fatigue severity scale, *SF-36vs* Vitality subscale of the MOS 36-item short-form health survey, *HCS* Healthy control subjects.

For all three fatigue instruments used, fatigue scores were significantly higher in patients than in healthy subjects. The median (IQR) fVAS-score was 43.0 (18.0–64.5) in patients, and 9.0 (2.0–16.0) in the control group (*p* < 0.001). Similarly, the FSS score was 3.8 (2.0–4.8) in patients, and 1.4 (1.0–1.9) in control subjects (*p* < 0.001); the inverted SF-36vs had a mean (SD) value of 58.8 (23.6) in patients, and 29.7 (14.3) in healthy subjects (*p* < 0.001); Fig. 2.

**Figure 3:**
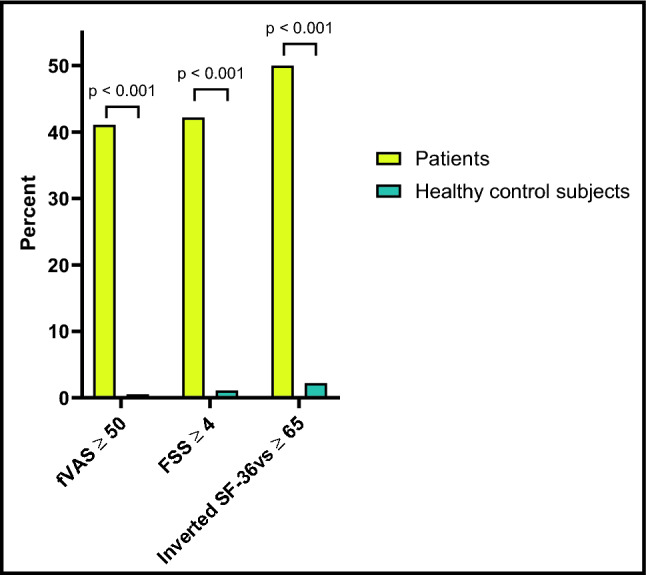
Prevalence of clinically relevant fatigue in 90 patients with newly diagnosed celiac disease vs. 90 healthy control subjects using defined cut-off values for fVAS, FSS and SF-36vs. Clinically relevant fatigue defined as: fVAS ≥ 50, FSS ≥ 4, and inverted SF-36vs ≥ 65. *fVAS* Fatigue visual analog scale, *FSS* Fatigue severity scale, *SF-36vs* Vitality subscale of the MOS 36-item short-form health survey.

Based on the predefined cut-off values for clinically relevant fatigue as recorded by the fVAS, FSS, and SF-36vs, the prevalence of fatigue in patients with celiac disease was 41.1%, 42.2%, and 50.0%, respectively, versus 0%, 1.1%, and 2.2% in healthy control subjects (all *p* < 0.001); Fig. 3.

There was no difference in fatigue severity when comparing patients with concurrent autoimmune disease to patients with only celiac disease (Supplementary Table 1).

### Factors associated with fatigue

In univariable regression analysis, age, female sex, inverted SF-36 pain, and HADS-D scores were significantly associated with fVAS, FSS, and inverted SF-36vs scores. Age was negatively associated with fVAS: β-0.318 (*p* = 0.002), FSS: β-0.311 (*p* = 0.003), and inverted SF-36vs scores: β-0.378 (*p* < 0.001). Sex (0 = female, 1 = male) was negatively associated with fVAS: β-0.323 (*p* = 0.002), FSS: β-0.371 (*p* < 0.001), and inverted SF-36vs: β-0.273 (*p* = 0.009), meaning that female sex was associated with fatigue. Pain and depression scores were positively associated. The associations between pain (inverted SF-36 pain) and fatigue were: fVAS: β 0.335 (*p* = 0.001), FSS: β 0.351 (*p* = 0.001), and inverted SF-36vs scores: β 0.334 (*p* = 0.001). The β-values reflecting the association between depression and fVAS, FSS, and inverted SF-36vs were 0.504 (*p* < 0.001), 0.501 (*p* < 0.001), and 0.334 (*p* = 0.001), respectively. BMI, cobalamin, folic acid, 25-OH vitamin D, hemoglobin, ferritin, tTG-IgA, and “any other autoimmune disease” showed no associations with fVAS, FSS, or inverted SF-36vs (Supplementary Table 2).

In multivariable regression analysis, both forward and backward selections resulted in the same model; this demonstrated a positive association between female sex, higher HADS-D-scores, higher inverted SF-36 pain scores, and fVAS, FSS, and inverted SF-36vs. Conversely, a negative association was shown between age and fVAS, FSS, and inverted SF-36vs, indicating that younger patients, females, and patients with more pain and/or more depression experienced more fatigue (Table 3).

**Table 3:** Final multiple regression models showing associations between fVAS, FSS, inverted SF-36vs, and selected clinical variables in 90 patients with newly diagnosed celiac disease.

Variables	fVAS		FSS		Inverted SF-36vs	
	β	*p* value	β	*p* value	β	*p* value
Age	− 0.263	0.002	− 0.252	0.002	− 0.327	< 0.001
HADS-D score	0.412	< 0.001	0.398	< 0.001	0.414	< 0.001
SF-36 Pain (inverted)	0.197	0.022	0.218	0.01	0.189	0.027
Male sex	− 0.269	0.001	− 0.319	< 0.001	− 0.215	0.009
R square for model	0.444		0.472		0.453	

*FSS* Fatigue severity score, *fVAS* Fatigue visual analog scale, *HADS-D* Hospital anxiety and depression questionnaire; depression subscale score, *Inverted SF-36vs* Inverted vitality subscale of the MOS 36-item short-form health survey, *SF-36 Pain* Pain subscale of the MOS 36-item short form health survey.

With regard to other independent values included in the multiple regression model selection, tTG-IgA did not contribute significantly to the fVAS score; BMI, hemoglobin, and tTG-IgA did not contribute significantly to the FSS score; and folic acid and tTG-IgA did not contribute significantly to the inverted SF-36vs scores. These variables were thus excluded from the final multiple regression models. The final model explained 44%, 45%, and 47% of the variance in the fVAS, SF-36vs, and FSS scores, respectively.

## Discussion

This study clearly demonstrates that fatigue is a common and substantial complaint in patients with newly diagnosed and untreated celiac disease. The findings were consistent across three different and widely used fatigue instruments, revealing that the prevalence of clinically relevant (meaningful) fatigue ranges between 41 and 50% in these patients. These results are in accordance with previous studies that describe more fatigue in patients with celiac disease, compared with healthy control subjects^37,38^.

The fatigue prevalence rates are similar to data reported in other chronic inflammatory conditions such as inflammatory bowel disease, primary Sjögren’s syndrome, and psoriasis^3,5,36,39^. This supports the hypothesis that fatigue represents a common mechanism across different conditions, and is triggered by chronic inflammation and immunological danger signals. Data regarding fatigue in celiac disease is scarce, and previous studies reporting prevalence rates of fatigue in celiac disease patients have not used validated fatigue instruments or defined cut-off values. Most previous studies reporting the prevalence of fatigue have used a simple question asking patients whether or not they suffered from fatigue^21,40–47^; only one study applied a disease-specific fatigue instrument (Celiac Disease Patient Reported Outcome, Non-gastrointestinal domain)^48^. The inconsistencies in rating and reporting could thus be a reason for the large variations and discrepancy in previous studies, ranging from 8 to 82%^24,25^.

Measuring fatigue is a challenge. To the best of our knowledge, our study is the first to apply multiple generic and unidimensional fatigue instruments, as well as use defined cut-off values, to rate clinically relevant fatigue in newly diagnosed patients with celiac disease. Generic fatigue instruments were chosen, as they are not influenced by disease-related factors; disease-specific fatigue instruments tend to comprise elements of disease activity or other disease features, not solely those related to fatigue. Generic instruments are also of value when aiming to compare fatigue between sick and healthy individuals, as well as between diseases.

Whether fatigue can be considered a unidimensional phenomenon, or whether there are several dimensions, such as mental fatigue, physical fatigue, and muscular fatigue, remains under debate. This is reflected in the numerous different fatigue questionnaires available. According to the latter comprehension, fatigue is a phenomenon that specifically influences cognition, muscles, mood, physical activity, etc., therefore representing separate features. However, we hypothesize that fatigue, in the context of sickness behavior, should be considered a universal phenomenon that influences different aspects of human life, including traits or attributes associated with the disease.

In this study, we used a single-item unidimensional instrument (fVAS), and two multi-item unidimensional instruments (FSS, SF-36vs). The consistency of the results indicates that the findings derived from the selected fatigue instruments are representative.

Several factors may influence fatigue severity; in our study, female sex and younger age were associated with fatigue. Previous studies on other diseases and conditions have been inconsistent regarding these items; and even though a statistically significant association was found in our study, it is not possible to make a generalized and universal conclusion^5,49–53^. However, the association between pain and fatigue has been consistent in many studies^15,54^. Pain elicits signals of bodily damage, and is an inducer of the sickness behavior response that is evolutionarily believed to increase the survival of the individual^55^; fatigue is a major component of this response^15^. A genome-wide association study (GWAS) of patients with Sjögren’s syndrome identified an association with genetic variants of the *RTP4* gene influencing the severity of fatigue. This gene encodes a protein involved in pain processing and points to a possible molecular explanation for the association^15,56^.

Depressive mood is a consistent factor associated with fatigue in chronic inflammatory conditions^3,5^. In addition, this association has been described in both treated and untreated patients^37,57^. Both depression and fatigue are elements in the sickness behavior response and are possibly signaled by common inflammatory and neurotransmitter pathways^58,59^. Some studies of people with mental depression show increased proinflammatory cytokine profiles^60^. The role of IL-1β in depression has been increasingly emphasized, and could be key to elucidating why fatigue and depression are closely associated^61^. An additional explanation is that the instruments used to assess fatigue and depression often have similar wording, therefore capturing elements of both dimensions. Finally, fatigue can also be a part of depressive disease entities with overlapping symptomatologies^62^. Although fatigue in celiac disease have been scarcely explored previously, it is likely that the mechanisms that generate fatigue are identical across chronic inflammatory diseases.

Anemia, as well as vitamin D, folic acid, cobalamin, and iron deficiencies, frequently occur in celiac disease^63–65^; however, we could not reveal any association between hemoglobin and fatigue, an observation possibly owing to the low number of patients with anemia (n = 5). The evidence regarding vitamin D deficiency and fatigue is conflicting; no associations were observed. The rates of cobalamin and folic acid deficiencies were too small for adequate analysis, and no associations with fatigue were observed.

This study has some limitations; first, we did not record sleep disturbances. Reduced sleep quality, as recorded by the Pittsburgh Sleep Quality Index, has been previously shown to influence fatigue in patients with celiac disease and IBD^38,49^. Another limitation is the cross-sectional study design, which cannot point to causality and only describes associations. Even though we selected predefined cut-off values that have been used in other studies to define fatigue prevalence, the validity of these cut-off values is debatable; conceptually it is artificial to dichotomize a continuous trait into “fatigue” or “no fatigue”.

In conclusion, patients with newly diagnosed celiac disease had significantly increased and more prevalent fatigue when compared with matched, healthy control subjects. Fatigue in these patients was associated with female sex, younger age, pain, and depressive mood. This confirms that fatigue is a substantial ailment in untreated celiac disease that deserves increased attention in the management of these patients.

## Supplementary Information

Supplementary Information.

## Abbreviations

BMI: Body mass index
FSS: Fatigue Severity Scale
fVAS: Fatigue Visual Analog Scale
GWAS: Genome-wide association study
HADS-D: The Hospital Anxiety and Depression Scale, Depression Subscale
Hb: Hemoglobin
HCS: Healthy control subjects
H&E: Hematoxylin and eosin
IL: Interleukin
SF-36 Pain: Pain subscale of the Medical Outcomes Study 36-Item Short-Form Health Survey
SF-36vs: Vitality subscale of the Medical Outcomes Study 36-Item Short-Form Health Survey
tTG-IgA: Anti-tissue transglutaminase-IgA antibodies
