# Supplementary material for: Fatigue: a frequent and biologically based phenomenon in newly diagnosed celiac disease
Source: Sci Rep. 2022 May 4;12:7281. doi: 10.1038/s41598-022-11802-8 (PMC9068783; doi:10.1038/s41598-022-11802-8)
Supplement: Supplementary file 1 — Supplementary Information. [file 41598_2022_11802_MOESM1_ESM.docx]

**Fatigue: a frequent and biologically based phenomenon in newly diagnosed celiac disease**

*Berit Mære Skjellerudsveen, M.D. ₐ, Roald Omdal, M.D., PhD _ae,_ Anne Kristine Hetta, M.D. ₐ, Jan Terje Kvaløy, PhD _bc,_ Lars Aabakken, M.D., PhD _d_, Inger Marie Skoie, M.D. _f_, Tore Grimstad, M.D., PhD _ae_*

*_a_ Department of Internal Medicine, Stavanger University Hospital, Stavanger, Norway*

*_b_ Department of Mathematics and Physics, University of Stavanger, Stavanger, Norway*

*_c_ Department of Research, Stavanger University Hospital, Stavanger, Norway*

*_d_ Department of Transplantation Medicine, Rikshospitalet, Oslo University Hospital, Oslo, Norway*

*_e_ Department of Clinical Science, University of Bergen, Bergen, Norway*

*_f_ Department of Dermatology, Stavanger University Hospital, Stavanger, Norway*

E-mail: [berit.mere.skjellerudsveen@sus.no](mailto:berit.mere.skjellerudsveen@sus.no)

**Supplementary table 1**: Fatigue severity in patients with concurrent autoimmune disease vs. patients with celiac disease only

| **Fatigue instrument** | **Patients with only celiac disease (n=75)** | **p-value** | **Patients with concurrent autoimmune disease (n=15)** |
| --- | --- | --- | --- |
| FSS-score | 3.8 (2.0-4.7) | 0.931 | 3.0 (2.5-5.0) |
| fVAS | 45.0 (18.0-64.0) | 0.778 | 29.0 (23.5-59.0) |
| Vitality score (inverted) | 60.0 (42.5-75.0) | 0.778 | 65.0 (37.5-70.0) |

Numbers presented as median (IQR), Mann-Whitney U used for comparison between groups.

**Supplementary table 2 - Linear univariable and multivariable regression models**

**Table S2a** Associations between fatigue Visual Analogue Scale (fVAS) and selected clinical and biochemical variables in 90 patients with newly diagnosed celiac disease. R square for multiple regression model 0.444.

| **Variables** | Univariate regression | | Multiple regression | |
| --- | --- | --- | --- | --- |
|  | β | p-value | β | p-value |
| Age | -0.318 | 0.002 | -0.263 | 0.002 |
| BMI | -0.134 | 0.209 |  |  |
| Cobalamin | -0.064 | 0.551 |  |  |
| Ferritin | -0.064 | 0.572 |  |  |
| Folic acid | 0.095 | 0.375 |  |  |
| HADS-score | 0.504 | <0.001 | 0.412 | <0.001 |
| Haemoglobin | -0.067 | 0.535 |  |  |
| SF-36 Pain (inverted) | 0.335 | 0.001 | 0.197 | 0.022 |
| Sex | -0.323 | 0.002 | -0.269 | 0.001 |
| Tissue transglutaminase | -0.121 | 0.258 |  |  |
| Vitamine D  Any other autoimmune disease | -0.068  -0.039 | 0.523 0.716 |  |  |
|  |  |  |  |  |

**Table S2b** Associations between Fatigue Severity Score (FSS) and selected clinical and biochemical variables in 90 patients with newly diagnosed celiac disease. R square for multiple regression model 0.472

| **Variables** | Univariate regression | | Multiple regression | |
| --- | --- | --- | --- | --- |
|  | β | p-value | β | p-value |
| Age | -0.311 | 0.003 | -0.252 | 0.002 |
| BMI | -0.193 | 0.068 |  |  |
| Cobalamin | -0.068 | 0.523 |  |  |
| Ferritin | -0.066 | 0.558 |  |  |
| Folic acid | 0.112 | 0.293 |  |  |
| HADS-score | 0.501 | <0.001 | 0.398 | <0.001 |
| Haemoglobin | -0.192 | 0.167 |  |  |
| SF-36 Pain (inverted) | 0.351 | 0.001 | 0.218 | 0.01 |
| Sex | -0.371 | <0.001 | -0.319 | <0.001 |
| Tissue transglutaminase | -0.074 | 0.491 |  |  |
| Vitamine D | -0.029 | 0.787 |  |  |
| Any other autoimmune disease | 0.009 | 0.934 |  |  |
|  |  |  |  |  |
|  |  |  |  |  |

**Table S2c** Associations between inverted SF-36 vitality score and selected clinical and biochemical variables in 90 patients with newly diagnosed celiac disease. R square for multiple regression model 0.453

| **Variables** | Univariate regression | | Multiple regression | |
| --- | --- | --- | --- | --- |
|  | β | p-value | β | p-value |
| Age | -0.378 | <0.001 | -0.327 | <0.001 |
| BMI | -0.107 | 0.317 |  |  |
| Cobalamin | -0.035 | 0.743 |  |  |
| Ferritin | -0.086 | 0.446 |  |  |
| Folic acid | 0.193 | 0.069 |  |  |
| HADS-score | 0.502 | <0.001 | 0.414 | <0.001 |
| Hemoglobin | -0.136 | 0.203 |  |  |
| SF-36 Pain (inverted) | 0.334 | 0.001 | 0.189 | 0.027 |
| Sex | -0.273 | 0.009 | -0.215 | 0.009 |
| Tissue transglutaminase | -0.097 | 0.362 |  |  |
| Vitamine D  Any other autoimmune disease | -0.017 -0.040 | 0.871 0.706 |  |  |
|  |  |  |  |  |

BMI = Body mass index; HADS-D = Hospital Anxiety and Depression questionnaire, depression subscale score; SF-36 Pain = Pain subscale of the MOS 36-Item Short Form Health Survey; tTG-IgA = Anti-Tissue Transglutaminase IgA antibodies.
Linear univariable and multivariable regression models with fVAS, FSS and inverted SF-36 VS as dependent variables.
